# Severe open Lisfranc injuries: one-stage operation through internal fixation associated with vacuum sealing drainage

**DOI:** 10.1186/s13018-016-0471-1

**Published:** 2016-11-04

**Authors:** Wenqing Qu, Shuqin Ni, Zhenhai Wang, Yong Zhao, Shimin Zhang, Yiheng Cheng, Tong Liu, Min Yu, Dan Wang

**Affiliations:** 1 Department of Orthopaedics and Trauma, Yantaishan Hospital, Yantai, 264008 China; 2 Department of Orthopaedics, Yangpu Hospital of Tongji University, Shanghai, 200090 China

**Keywords:** Lisfranc joints injury, Openness, k-wire internal fixation, VSD

## Abstract

**Background:**

This study aimed to investigate the clinical feasibility of treating severe open Lisfranc injuries by means of one-stage internal fixation with k-wires associated with vacuum sealing drainage (VSD).

**Methods:**

The clinical outcomes of 20 cases of severe open Lisfranc joint fracture-dislocation treated by using one-stage internal fixation with k-wires associated with VSD, after debridement and suturing during emergency treatment, were reviewed.

**Results:**

At 6 and 12 months after surgery, the American Orthopaedic Foot and Ankle Society midfoot scores were 69.2 and 78.2, the positive rates were 75 and 85 %, and the average visual analogue scale scores were 4.3 and 1.3, respectively. The average time of internal fixation surgery was 47 min (30–70 min). There were three cases of wound-edge necrosis; however, there were no cases of skin necrosis around the incision, or deep infection. The mean time of first hospital stay was 16.1 days (10–23 days).

**Conclusions:**

Treatment of severe open Lisfranc fracture and dislocation through one-stage internal fixation with k-wires in association with VSD led to fast anatomical reduction, stabilized bony structure, fast soft tissue recovery, and good short-term follow-up results.

## Background

High-energy open Lisfranc injuries mainly result from being crushed under a heavy object, traffic accidents, or falling from a height and are represented by severe Lisfranc joint fracture-dislocation with serious soft tissue injuries. Improper treatment of these fractures might lead to negative outcomes such as soft tissue necrosis, posttraumatic arthritis, and arch abnormalities [1]. Staged treatment has been widely applied in clinical practice; however, many patients were hospitalized for a long time with unsatisfactory clinical outcomes, such as poor recovery of soft tissue after temporary fixation with an external fixator, leading to a delay in performing open reduction internal fixation (ORIF) in the second stage, resulting in difficulty or even failure in performing ORIF [2–5]. From March 2011 to January 2015, 30 cases of severe open Lisfranc injuries were treated with one-stage k-wire internal fixation associated with vacuum sealing drainage (VSD) at the Foot and Ankle Surgery Department of our hospital, with satisfactory outcomes. In this report, 20 cases with intact information are summarized and analyzed.

## Methods

### General data

This study included 17 male and 3 female patients with a mean age of 49.5 years (range, 22–65 years). According to the Gustilo classification for open injuries, there were 7 type II cases and 13 type III cases. According to the Myerson classification for Lisfranc injuries, there were 4 type B2 cases, 4 C1 cases, and 12 C2 cases. All injuries were unilateral. The causes of injury were as follows: being crushed under a heavy object, 11 cases; traffic accident, 5 cases; and fall from a height, 4 cases. Anteroposterior, lateral, and oblique radiographs were obtained for all patients before surgery. Computed tomography (CT) scanning and three-dimensional reconstruction were performed in nine cases. There were two cases with combined phalangeal fracture, five cases of articulatio tarsi transversa or articulatio cuneonavicularis fracture and dislocation, two cases of ankle fracture, one case of spinal fracture, and two cases of rib fracture. After excluding any contraindication, emergency surgery was performed in all cases, and the average injury-to-surgery interval was 4.4 h (2–13 h). This study was conducted in accordance with the Declaration of Helsinki and with approval from the Ethics Committee of Yantaishan Hospital. Written informed consent was obtained from all patients.

### Surgical methods

Preventative antibiotics were intravenously given to patients 30 min before surgery. Eleven patients were anesthetized with spinal or epidural anesthesia, and seven patients with general anesthesia with tracheal intubation. The patients were laid in the supine position. A tourniquet was applied to the proximal end of the thigh. The wound was washed first with hydrogen peroxide and then with a large amount (>3000 mL) of normal saline (NS). The wound, including the leg from below the knee, was soaked in povidone-iodine, followed with routine draping. Completely detached bone blocks were also soaked in povidone-iodine for further surgery. In 18 cases, the open wound was located at the dorsum. Wound debridement was performed from the surface to the deeper part, leaving as much skin tissue as possible, including peeled skin. Contused and devitalized soft tissue such as subcutaneous tissue, fascia, and tendon sheath were removed, whereas large bone blocks, but not small fragments, were kept. Important muscle tendons, blood vessels, and deep and superficial peroneal nerves were marked. Then, the surface was again washed repeatedly with hydrogen peroxide, povidone-iodine, and NS.

One-stage internal fixation with k-wires was performed as definitive fixation for osteoarticular injury. Reduction was performed through the original wound in ten cases. In eight cases with Myerson type C injuries, an extra incision was made to perform anatomical reduction under direct vision. Reduction started from the cuneiforms to the base of metatarsals, from the medial, to central, to lateral columns. The mortise and tenon structure, formed by the cuneiforms and the base of the second metatarsal, and other undamaged bones were used as anatomical markers. To maintain the reduction, the bones were clamped with towel forceps. The bones and joints were fixed by using the k-wires through the wound or by penetrating the skin, as follows: in 1st ray, at least two 2.0-mm intersecting k-wires were used for stable fixation; in ray 2–4, a 1.5-mm k-wire was used for vertical fixation respectively; and in ray 5, another 2.0-mm k-wire was used. Extra intersecting fixation was provided horizontally, if necessary, through the base of metatarsals or the cuneiforms to achieve global Lisfranc stability. Anteroposterior and lateral radiographs were taken to examine the positional relationship between the base of the first and second metatarsals and the medial and intermediate cuneiforms, respectively, and 30° internal oblique radiographs were obtained to examine the positional relationship between the base of the fourth metatarsal and the medial border of the cuboid. If the bones could not be accurately restored, reduction of midfoot malalignment was prioritized.

**Fig. 1:**
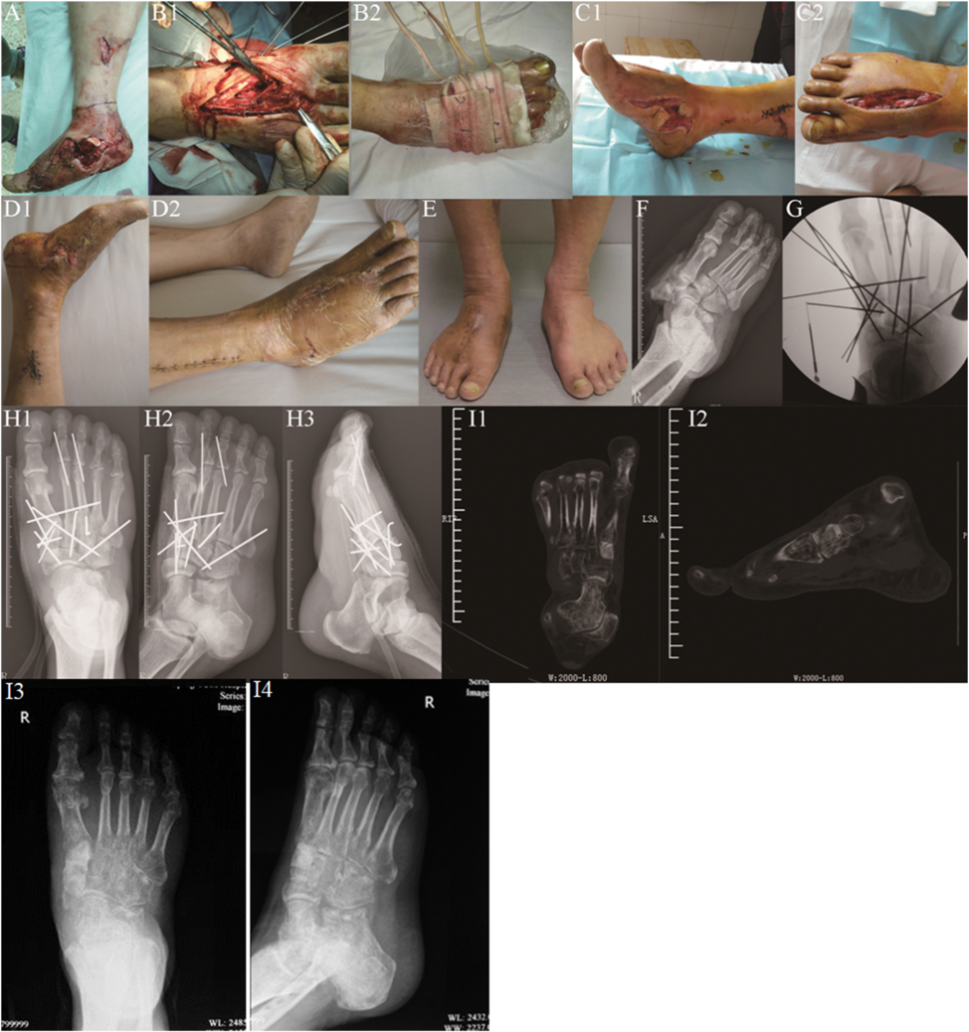
Male patient, aged 55, with right foot severe open Lisfranc fracture-dislocation, was admitted to hospital 2 h after being crushed under heavy objects. One-stage internal fixation using k-wires associated with VSD was performed during emergency. **a** Severe open wound on the right foot with Lisfranc fracture-dislocation and badly contaminated, combined with ankle fracture shown on X-ray film. **b** One-stage reduction and multiple k-wire internal fixation were performed via the open wound and dorsal incision, sealed with VSD kit. **c** The condition of original wound and incision was checked when VSD kit was changed 5 days after surgery. **d** The patient was discharged from hospital 23 days after surgery, when the dorsal incision recovered well after suture, and the original open wound also recovered well after postage stamp grafting. **e** One year after injury and 8 months after weight-bearing walking, with satisfying appearance and function. **f** On X-ray film before surgery, fracture-dislocation was observed to the right Lisfranc joint, combined with distal fibula fracture. **g** Multiple k-wire cross-fixation was performed during surgery; the fracture-dislocation reached to anatomical reduction under X-ray. **h** AP, oblique, and lateral X-ray showing the condition of fracture-dislocation after one-stage internal fixation with good alignment. **i** Space broadening was not observed by CT review and X-ray film half a year after removing internal fixators

**Fig. 2:**
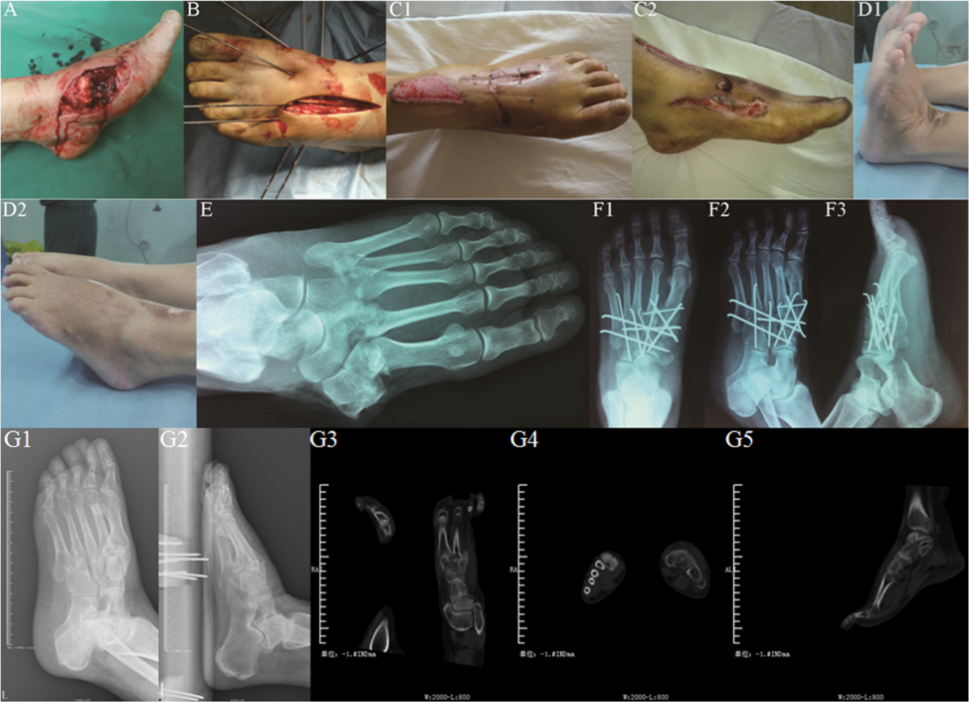
Female patient, aged 52, with left foot severe open Lisfranc fracture-dislocation, was transferred to our hospital 3 h after traffic injury. One-stage internal fixation using k-wires associated with VSD was performed during emergency. **a** Severe open wound on the left foot with bone exposure. **b** One-stage reduction and multiple k-wire internal fixation were performed via the open wound and dorsal incision, sealed with VSD kit. **c** The condition of original wound and incision was checked when VSD kit was changed 4 days after surgery. **d** Four months after surgery when the internal fixators were removed, with satisfying appearance and function. **e** On X-ray film before surgery, fracture-dislocation was observed at the left Lisfranc joint. **f** AP, oblique, and lateral X-ray showed that the fracture-dislocation reached to anatomical reduction after one-stage internal fixation. **g** X-ray film and CT scan showed good matching midfoot joint after removing the internal fixation at half a year operation

To close the wound, the tourniquet was loosened and a new incision was performed with interrupted, low-tension suture or exclusion after hemostasis. A low-tension suture was also performed on the original wound with severe skin retraction or peeling, with the application of several interrupted stitches to draw the skin margin together, facilitate drainage, and avoid further skin necrosis. VSD accessories (Wuhan VSD Medical Science & Technology Co. Ltd., Wuhan, China) were used to cover the wound, seal the main film, connect the vacuum pump, and start suction (Figs. 1 and 2).

### Treatment after surgery

The patients were given routine antibiotics to prevent infection. The VSD kit was opened to evaluate wound surface recovery after 5–7 days of suctioning. Another debridement, skin graft, or skin flap surgery was performed on patients with skin or soft tissue coloboma or new skin necrosis after the initial debridement. After the surgery, VSD was continued, and another evaluation and wound treatment were done after another 7 days. The k-wires through the lateral column were removed in the outpatient clinic after 6–8 weeks, and the k-wires through the medial and central columns were removed in the ward after 3–4 months. Partial-weight-bearing walking and active and passive joint function training of the foot and ankle were started after removing all internal fixators. The schedule for full-weight-bearing walking depended on the radiographic findings (usually at 8 weeks after the removal of internal fixators).

The radiographs of the patients (some of the patients were evaluated with CT) were reviewed at 6 weeks, 12 weeks, 6 months, and 1 year after the initial surgery. Moreover, functional scoring was performed by using the American Orthopaedic Foot and Ankle Society (AOFAS) score.

## Results

### Surgery details

The average time of debridement, internal fixation, and VSD wound closing was 47 min (30–70 min). The average number of k-wires for each case was 7.4 (5–13), and a total of 62 2.0-mm and 75 1.5-mm k-wires were used. The original open wound was completely closed in stage 1 in six cases, sutured in stage 2 in eight cases, recovered after skin graft in five cases, and repaired with skin flap in one case. The extra incision made during surgery (in eight cases in total) was completely closed in stage 1 in six cases and sutured in stage 2 in two cases. VSD was used for 47 times in all cases. Because the front foot was not bearing load before the k-wires were removed, the k-wires did not lead to serious soft tissue irritation. The internal fixators were removed in stages after surgery. The fixators through the lateral column were removed after 6–8 weeks, and those through the medial and central columns were removed after 10–16 weeks.

### Follow-up

The average follow-up time for 18 cases was 16.4 months (12–26 months). No patient developed osteofascial compartment syndrome, and no loosening or cracking of the internal fixator was observed in any cases. Radiographic examinations immediately and at 6 weeks, 12 weeks, 6 months, and 1 year after surgery indicated that the average recovery time for bone fracture was 12 weeks (8–20 weeks). In two cases with cuneiform coloboma, autologous iliac crest graft was performed 12 weeks after the initial internal fixation, which healed after 20 weeks. Spontaneous partial joint fusion was observed in two cases, and wound-edge necrosis occurred in three cases; however, there were no cases of necrosis of the incision edge, or deep infection. The mean time of first hospital stay was 16.1 days (10–23 days).

The AOFAS midfoot scoring system was applied for functional evaluation at 6 and 12 months after surgery. The average scores at 6 and 12 months were 69.2 (55–86) and 88.2 (68–95), the positive rates were 75 and 85 %, and the average visual analogue scale scores were 4.3 (2–6) and 1.3 (0–3), respectively.

In four cases, loading resulted in foot pain in the lower arch. The symptoms were improved after a customized insole treatment, and the patients did not undergo joint fusion surgery.

## Discussion

Lisfranc injuries are often difficult to diagnose and treat, causing long-term disability without appropriate management [6–8]. The difficulty in the clinical treatment of open Lisfranc fracture and dislocation results from the severe destruction of the bony structure, with poor condition of local soft tissue. Thus, staged treatment has been frequently performed [2–5], in which general alignment and restoration of the length of the foot are first performed by using temporary external fixators, facilitating soft tissue recovery, and then a final definitive internal fixation or joint fusion is performed after the body and local soft tissue condition has improved. This method has been widely applied in the treatment of complex tibial plateau and pilon fractures, with good results. However, in many cases with severe Lisfranc joint fracture and dislocation, especially those with open wound, the long recovery time for soft tissue in stage 1 delayed the internal fixation in stage 2. Sufficient incision for exposing the injury area was not allowed owing to the soft tissue condition of the newly healed wound or fracture-dislocation area after skin graft or skin flap coverage, and the formed cicatrix and inflammatory granulation tissue caused difficulties in reduction during surgery. Application of an internal fixation plate, which is firm but large, also required a good soft tissue condition. Moreover, in some cases, ORIF could not be performed, which could only be remedied with osteotomy or fusion surgery. In one study, 123 cases with staged treatment for high-energy midfoot fractures-dislocations, with an average time of 21.3 days for the application of external fixators, were reported, similar to our previous clinical experience [2]. Therefore, it is practical to attempt a safe and effective one-stage definitive internal fixation method.

### Soft tissue management

Satisfactory results can be achieved with open reduction for Lisfranc injuries. However, despite this treatment, both the severity of the soft tissue injury and nonanatomic reduction are negative prognostic factors in the treatment of Lisfranc fractures-dislocations [9].

Soft tissue treatment is especially important for open Lisfranc fracture-dislocation. VSD has been applied in many cases, leading to earlier wound closure, clear wound surface drainage, faster detumescence, accelerated tissue growth, and reduced clinical workload, compared with traditional methods [10, 11]. Moreover, wounds in most cases showed good improvement when the VSD accessory was changed for the first time at 5–7 days after surgery, and soft tissue was fast repaired simply with direct suture, skin graft, or skin flap transplantation, owing to emergency measures such as washing with a large amount of NS, keeping as much skin as possible, avoiding high-tension suture, and drilling on the exposed bone with a thin k-wire.

### Final definitive internal fixation with one-stage anatomic reduction and k-wires

Nithyananth performed this treatment in 22 cases and obtained satisfactory function scores during long-term follow-ups with 16 days of average wound healing time, much shorter than our experience before using an external fixator (21.3 days). The exposure, reduction, and fixation of severe midfoot fracture-dislocation were normally performed through a dorsal approach: with high bone density, no obvious bone coloboma after severe bone injury was fixed with k-wires; the reduction of adjacent joints can help estimate the reduction degree of target joints as they wedged with each other. The characteristics of the soft tissue of this area allow extra auxiliary incision as the tolerance of the tissues to violent injuries such as grinding or tearing was high; for instance, midfoot skin and subcutaneous tissue gain tolerance from long-term friction and extrusion from shoes, as well as the ligament of joint dorsum and extensor digitorum longus tendon, but there was hardly any muscle tissue with low tolerance. Most of the fracture-dislocation area could be exposed through the open wound, as this injury was superficial when surgery was performed through the dorsal approach, or anatomical reduction could be performed under direct vision through auxiliary incision, without increased risk of skin necrosis. The k-wire was small enough for cross-fixation for providing high strength, particularly when three to four 2.0-mm k-wires were applied to the medial and lateral columns to form a “splint-like” fixation for the central column. Thinner k-wires were applied to the central column to decrease the damage area of the articular cartilage surface and reduce bone infection risk significantly after surgery, compared with that using a screw spike or fixation plate.

Anatomical reduction is the basic principle of treating intra-articular fracture-dislocation. Many researchers indicated that rigorous anatomical reduction should be performed, as functional recovery after surgery directly relates to reduction, regardless of performing a true percutaneous technique or open methods [9, 12–14]. Previous studies demonstrated that malreduction during surgery led to poor function and imaging scores in up to 49.6 % cases [15, 16]. Nithyananth, Kadow, and Kuo demonstrated the standard to ensure anatomical reduction during surgery [1, 2, 17]: the tala axis and the first metatarsal axis were nearly overlapping on the foot anteroposterior film (the average angle between the two was 7.7°). The medial and lateral borders of the first metatarsal, and the medial and lateral borders of the medial cuneiform, respectively, were in one line, as were those of the second metatarsal and the intermediate cuneiform, forming an evenly matched cross-joint space, with the width between the medial cuneiform and medial border of the second metatarsal being <2 mm. On a 30° internal oblique radiograph, the medial border of the fourth metatarsal bone and that of the cuboid were in one smooth line, and the calcaneus axis and the fourth metatarsal axis were overlapping. On the lateral film, no metatarsal shift was observed toward the metatarsal side or the dorsal side, and no collapse arch or high-arch deformity was observed; the talo-first metatarsal angle (Meary’s angle) was between ±4° [18]. If anatomical reduction was difficult to perform because of severe fracture, alignment recovery of the midfoot and hindfoot was prioritized.

### Comparison of the therapeutic strategy between one-stage internal fixation and joint fusion

Midfoot fracture-dislocation has been treated with one-stage joint fusion, based on the theory that the medial column is responsible for stabilization and support, and the lateral column for flexibility and cushioning stress [19]. The method by Boffeli was the most typical, in which one-stage fusion was performed in the medial column, ORIF in the central column, and k-wire fixation in the lateral column [20]. Some researchers demonstrated no statistically significant difference in the functional scoring and satisfaction degree between cases treated with one-stage joint fusion and those treated with one-stage internal fixation, after a long-term follow-up [14, 19, 21]. The advantage of joint fusion was that the probability of removing the internal fixator was low, whereas the disadvantages were as follows: joint fusion requires skilled doctors and is difficult to fulfill during emergencies or hospital night shift, and fixation in standard joint fusion by using a screw spike through a joint or a fixation plate across a joint significantly increases the risk of bone infection in cases with open injury. After medial column fusion of Lisfranc joints, the load of adjacent joints, such as the central column, metatarsophalangeal joints, and transverse tarsal joint, will increase, resulting in posttraumatic arthritis.

In our study, Lisfranc posttraumatic arthritis was identified on the radiographs of seven cases 1 year after surgery, with mild clinical symptoms. Similar results showing that arthritis observed on radiographs was not in accordance with clinical symptoms were also reported in another study after a long-term follow-up [16]. We speculated that the medial and central columns of Lisfranc joints are amphiarthrodial joints with low abrasion. Although the strength of internal fixation with k-wires is lower than that with a fixation plate, joint stability was enhanced as extensive soft tissue injury generated large amounts of fibrous cicatrices around the joints during recovery, and ankylosis, not arthrochalasis, occurred after surgery, similar to subtalar joint ankylosis that occurs after treatment of calcaneal fractures by means of internal fixation with a steel plate through an L-shaped calcaneal lateral incision. In the cases in our short-term study, space broadening was not observed in the joints with the original injury on CT review observed after 6 months of full-weight-bearing walking (Fig. 1h); however, long-term follow-up of joint stability requires further investigation. It can be speculated that although symptomatic Lisfranc instability, fallen arches, or traumatic flat foot was observed after surgery, this can be treated by using customized shoe pads or by performing joint fusion after the soft tissue has fully recovered. Thus, except for joint dislocations without fracture, we prefer one-stage internal fixation for Lisfranc fracture-dislocation over joint fusion.

## Conclusions

In conclusion, the goal of the treatment of Lisfranc fracture-dislocation is to achieve a painless, functional plantigrade foot with a good appearance [22]. Although staged treatment for severe open Lisfranc fracture and dislocation has been frequently reported and has shown acceptable results, one-stage internal fixation with k-wires associated with VSD led to faster anatomical reduction, stabilized bony structure, faster soft tissue recovery after surgery, and good short-term follow-up results according to our research, although the medium- and long-term follow-up results concerning joint stability require further investigation.

## Abbreviations

AOFAS: American Orthopaedic Foot and Ankle Society
CT: Computed tomography
NS: Normal saline
ORIF: Open reduction internal fixation
VSD: Vacuum sealing drainage
